# Skinfold Thickness as a Cardiometabolic Risk Predictor in Sedentary and Active Adult Populations

**DOI:** 10.3390/jpm13091326

**Published:** 2023-08-29

**Authors:** Sughey González-Torres, Luis Miguel Anaya-Esparza, Gabriel Fermín Trigueros del Valle, Edgar Alfonso Rivera-León, Zuamí Villagrán, Sergio Sánchez-Enríquez

**Affiliations:** 1 Division de Ciencias Biomédicas, Centro Universitario de Los Altos, Universidad de Guadalajara, Rafael Casillas Aceves 1200, Tepatitlán de Morelos 47620, Mexico; sgonzalez@cualtos.udg.mx (S.G.-T.); edgar.rleon@academicos.udg.mx (E.A.R.-L.); 2 Division de Ciencias Agropecuarias e Ingenierias, Centro Universitario de Los Altos, Universidad de Guadalajara, Rafael Casillas Aceves 1200, Tepatitlán de Morelos 47620, Mexico; luis.aesparza@academicos.udg.mx; 3 Hospital Regional N°180, Instituto Mexicano del Seguro Social, Carretera San Sebastian-Santa Fe 1000, Tlajomulco de Zuñiga 45653, Mexico; gabriel.trigueros@imss.gob.mx

**Keywords:** body composition, cardiometabolic health, insulin resistance, blood pressure

## Abstract

Studies report that increased body fat can lead to health risks for individuals. However, some methods used for analyzing adiposity did not identify its distribution in the human body because they are typically measured using bioimpedance scales. This study aims to associate the presence of cardiometabolic risk factors in sedentary and active adult populations through anthropometric methods based on skinfold thickness measurements. A cross-sectional study was conducted on 946 adults aged between 18 and 79 years with prior informed consent. Clinical, anthropometric, and biochemical parameters, as well as some cardiometabolic risk factors, were evaluated. Almost half of the population (45.1%; n = 427) is sedentary. A significant association was found between the sum of the skinfolds (bicipital, tricipital, subscapular, and suprailiac) and the cardiometabolic risk factors evaluated, highlighting the cardiovascular risk associated with abdominal obesity, risk of insulin resistance, as well as the development of hyperglycemia, and hypertriglyceridemia. The bicipital fold was thicker (19.67 mm) in the population with a sedentary lifestyle than in the physically active population (18.30 mm). Furthermore, the skinfolds that predict higher metabolic risks were suprailiac and subscapular in sedentary and active populations. Thus, these skinfold measurements could be considered in assessing the adult population for early cardiometabolic risk detection, even in healthy and physically active people.

## 1. Introduction

Sedentary lifestyles continue to prevail in the world’s population; more than 25% of the adult population does not perform enough physical activity, which is increasing in low-income countries [1]. The WHO defines a physically inactive/sedentary person as someone who engages in less than 150 min of moderate or 75 min of vigorous physical activity and remains seated for more than three hours per day. In this context, in the Mexican population, 29% of individuals perform less than 2.5 h of physical activity per week [2].

Adults with insufficient physical activity increase their risk of death by up to 30% compared to subjects who engage in at least 150 to 300 min of moderate exercise or 75 to 150 min of intense aerobic activity per week [1]. Several studies have demonstrated that physical inactivity promotes body adiposity [3,4,5]. It is well known that the accumulation of body fat, mainly in the abdomen, is strongly associated with the presence of cardiometabolic risks such as high blood pressure, insulin resistance, dyslipidemia, diabetes mellitus, and metabolic syndrome, among others [6,7,8], because visceral fat is an active metabolic tissue that modulates numerous adipocytokines associated with non-communicable chronic diseases [9].

Various methodologies have been reported for adiposity measurements, such as bioimpedance (determines total body water and fat-free mass by running a small electrical current through the body) and anthropometry (determines relative adiposity throughout measurement of weight, height, circumference, and skinfold thickness). These methods are reliable, simple, accessible, fast, noninvasive, and validated techniques used to analyze body composition. However, most of them exhibited limitations as they did not identify the fat distribution in the human body. Furthermore, skinfold thickness measurements could be used to estimate body density and corporal distribution of fat due to skinfolds not being altered by factors such as food intake, daily activity, or hydration status. [10]. Skinfold measurements (biceps, triceps, subscapular, and suprailiac) have been investigated for adiposity diagnosis in Spanish and Latin American children and adolescents [11]. Suprailiac and abdominal skinfold thickness were used to estimate body density in Japanese adults [12]. In addition, subscapular skinfold thickness has been associated with the incidence of type 2 diabetes mellitus in individuals from Peru [13]. It is also recognized that body fat distribution in individuals (not necessarily their total fat mass) is a factor that could promote cardiovascular diseases due to the expandability of subcutaneous adipose tissue [14]. Therefore, this study aimed to associate the presence of cardiometabolic risk factors in sedentary and physically active adult populations through anthropometric methods based on skinfold thickness.

## 2. Materials and Methods

The present epidemiological study is cross-sectional and descriptive. A non-probabilistic sampling method was used, incorporating the adult population of the metropolitan area of Jalisco, Mexico, with a 95% confidence interval using the statistical program Epi Info™ 7, through Statcalc (α = 0.3), where a minimum sample of 891 subjects was determined, and a total of 946 subjects consented to participate. The information was collected following the established ethical principles [15]. The project was approved by the ethics and research committee of the Unidad Clínica de Bioequivalencia S. de R.L. with registration number CONBIOETICA-14-CEI-002-2016718 and folio number 000622.

The assessment instrument included demographic factors, including sex, age, schooling, marital status, family and personal hereditary history, alcohol habits, smoking, and physical activity. Blood pressure was measured using a digital baumanometer (Omron^®^, Kyoto, Japan). Anthropometric measurements were performed using weight and fat percentages via impedance using a TBF-300A Body Composition Analyzer (Tanita^®^, Tokyo, Japan), height using a portable stadiometer SECA 213, and waist and hip circumferences using SECA 203 (SECA^®^, Hamburg, Germany). Additionally, to obtain bicipital, tricipital, suprailiac, and subscapular skinfold thicknesses, a Lange caliper was used (Lange^®^, California, USA) [16]. A sum of skinfolds was obtained to measure adiposity [17]. Metabolic risks were evaluated according to national guidelines [18], including abdominal obesity, waist-to-height ratio, insulin resistance, hyperglycemia, hypoalphalipoproteinemia, hypertriglyceridemia, and blood pressure, as shown in Table 1.

Regarding biochemical parameters and inflammation markers, blood samples were collected after subjects fasted for more than 8 h in dry vacutainer tubes and EDTA tubes (BD Vacutainer^®^, Sweden, Germany), and the sample was subsequently centrifuged (Eppendorf^®^, 5810R, Hamburg, Germany) at 3260× *g* for 15 min at 4 °C to separate the blood serum. Biochemical determinations were analyzed by the enzymatic colorimetric method (Biosystems^®^, Barcelona, Spain), while the ELISA technique (DRG International, Inc. Springfield, USA) was used to quantify insulin. All procedures were performed according to the supplier’s specifications.

Physical activity was determined using a short questionnaire, and subjects were classified as sedentary if they engaged in physical activity one or two times per week and as active if they engaged in physical activity at least one hour per day for five days.

The activity was classified according to intensity:

- Mild activity: subjects who exercised less than 60 min per day and fewer than 5 days per week.
- Moderate activity: subjects who exercised less than 90 min per day and fewer than 6 days per week.
- Intense activity: subjects who exercised more than 90 min and at least 6 days per week.

### Statistical Analysis

Data are presented as mean ± standard deviation. For statistical analysis, the normality (Kolmogorov–Smirnov test) and homeostacity (Levene’s test) were validated prior to data analysis. Student’s T-test was used to compare means by sex and physical activity. Subjects were distributed in quartiles according to their skinfold thickness. A comparison between quartiles was performed by the ANOVA test and Tukey´s post hoc. Additionally, chi-square and Cramer’s V tests were used to identify the association between skinfold thickness and cardiometabolic risks in active and non-active subjects. These statistical analyses were performed using SPSS v. 20 software (SPSS^®^ Statics, IBM^®^, Chicago, IL, USA). Values of *p* < 0.05 were considered statistically significant.

Subsequently, principal component analysis (PCA) was performed in two steps: the first analyzed the clusters for the variables of interest concerning sex and physical activity, and the second step comprised the specific analyses for the skinfolds (subscapular/suprailiac) that showed the strongest association with metabolic risks. In all cases, PCA was calculated without rotation, and the number of extracted factors was based on eigenvalues > 0.5 and explained variance > 90% using the Statistica software (v. 12 Stat Soft^®^, Tulsa, OK, USA).

## 3. Results

### 3.1. General Characteristics of the Population

The study was conducted at the Centro Universitario de Ciencias de la Salud (CUCS) of the University of Guadalajara. A total of 946 subjects participated in this study; 65% were women. The mean value indicated overweight according to the percentage of body fat (31.61%) coinciding with that stipulated by the body mass index (BMI of 27.55). Waist circumference values were found to be higher (men = 96.01 cm and women = 87.79 cm) than those considered normal for Mexican men (>90 cm) and women (>85 cm) [18] (Table S1).

The population consists of apparently healthy people with no previous knowledge of diagnosed chronic diseases, aged between 18 and 79 years (average age of 43.72), resulting in 72.5% (n = 681) of overweight or obese subjects. Regarding BMI, the population mean showed overweight subjects. Furthermore, the average values for blood pressure (systolic = 116.49 mmHg and diastolic = 75.58 mmHg), heart rate (68.96 beats/min), and respiratory rate (17.19 breaths/min) of the evaluated population were within normal parameters; as were the biochemical parameters evaluated that include hemoglobin (14 g/dL), hematocrit (43.82 mm/dL), glucose (94.94 mg/dL), total cholesterol (187.17 mg/dL), HDL cholesterol (42.97 mg/dL), LDL cholesterol (115.33 mg/dL), VLDL cholesterol (28.95 mg/dL), and triglycerides (144.98 mg/dL). Concerning the biomarkers of inflammation, the average C-reactive protein (1.95 mg/L) showed a slight increase in the values considered as medium cardiovascular risk, according to the Framingham scale.

Table 2 compares the following groups: female vs. male, female vs. female, and male vs. male. Each of the groups pertains to physical activity on anthropometric, clinical, and biochemical characteristics. In general, almost all evaluated parameters exhibited statistical differences (*p* < 0.05) between females and males (Table 2), excluding age, body mass index, heart and breathing rate, glucose, LDL cholesterol, fibrinogen, insulin, and ultrasensitive CPR values (*p* > 0.05). All skinfolds showed a higher thickness in females (bicipital = 20.83 mm, tricipital = 27.93 mm, suprailiac = 27.93 mm, and subscapular = 25.20 mm) than in males (bicipital = 15.35 mm, tricipital = 20.55 mm, suprailiac = 25.58 mm, and subscapular = 23.09 mm) with a marked difference in the bicipital and tricipital folds.

Regarding the comparison between physically active and non-active subjects in females, higher figures were found in sedentary women compared to active women in the parameters of weight, BMI, body fat percentage, waist, hip, and bicipital skinfold thickness. This is contrary to what was observed in male subjects, where the highest percentage of fat was reported in physically active men (26.09%) compared to non-physically active men (24.11%). However, the highest insulin levels were found in inactive men (Table 2).

**Figure 1:**
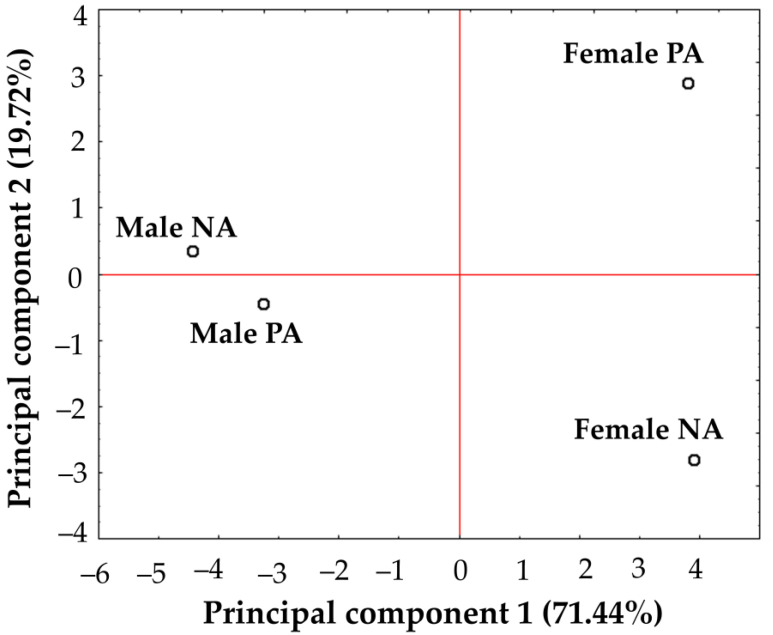
Principal component analysis. Distribution pattern of male and female by physical activity. PA: Physical Activity, NA: No Physical Activity. PCA was calculated without rotation, and the number of extracted factors was based on eigenvalues > 0.5 and explained variance > 90%.

Additionally, a principal component analysis (PCA) was performed on the behavior of variables related with physical activity and sex. Principal component 1 (PC1) and PC2 accounted for 91.16% of the total variance. The results showed a similarity between men regardless of their physical activity regime. On the other hand, there was a marked difference between physically active and sedentary women (Figure 1).

Body anthropometry was employed via the measurement of skinfold thickness. For this, a quartile classification was applied, considering quartile one as the lowest skinfold thickness and quartile four as the highest (Table 3). As the thickness of the skinfold sum increased, there was an increase in systolic pressure, glucose, LDL cholesterol, fibrinogen, insulin, fat percentage, and fat mass values. Moreover, statistical differences (*p <* 0.05) among quartiles were found for blood pressure, heart rate, respiratory rate, hemoglobin, hematocrit, glucose, LDL cholesterol, triglycerides, total leukocytes, fibrinogen, insulin, ultrasensitive CRP, fat percentage, and fat mass, suggesting that these parameters could vary, as skinfold thickness varies.

### 3.2. Metabolic Risks Concerning Skinfold Thickness

Participants who reported being sedentary constituted 45.1% (n = 427) of the evaluated population; of these participants, women reportedly made up 45.6% (n = 281) compared to men, who made up 44.2% (n = 146). Furthermore, participants who performed physical activity accounted for 54.9% (n = 519) of subjects, which were distributed as follows: light activity 32.7% (n = 108) vs. 21.4% (n = 132), moderate activity 19.7% (n = 65) vs. 24% (n = 148), and vigorous activity 3.3% (n = 11) vs. 8.9% (n = 55), of men and women, respectively.

Table 4 shows the association of metabolic risks regarding skinfold thickness in sedentary and active adults by Cramer´s V test. A sedentary lifestyle was reported in 45.1% (n = 427) of the population. Additionally, the sum of skinfolds was analyzed concerning the observed presence of metabolic risks in the population. A relationship (*p <* 0.001) was found between skinfold thickness and the presence of hyperglycemia, coronary risk (due to increased waist measurements), insulin resistance (by the HOMA-IR indicator), cardiometabolic risk (by the waist height index); hypertriglyceridemia, elevated blood pressure (*p >* 0.05), and a trend in decreased HDL cholesterol levels (*p* = 0.056).

When comparing the skinfold thickness of physically active subjects versus sedentary subjects, only the bicipital skinfold (18.30 vs. 19.67) showed statistical differences (*p <* 0.05). Furthermore, when skinfold thickness was individually associated with physical activity, a moderate association (*r*-value between 0.2 and 0.6) between cardiovascular risk (due to waist–height index) and skinfolds was found. In sedentary subjects, moderate associations were observed between the risk of chronic non-communicable diseases and hyperglycemia (elevated glucose) in the bicipital skinfold. In contrast, the suprailiac and subscapular skinfolds were associated with insulin resistance. The rest of the boxes marked with one asterisk also exhibited a weak association between the considered variables.

A positive correlation was found between skinfold thickness and cardiometabolic risks (Table 5). Cardiovascular risks were associated with abdominal obesity, waist-to-height ratio, insulin resistance, hyperglycemia, and C-reactive protein. On the other hand, a negative correlation between hemoglobin values and respiratory frequency in the bicipital and tricipital skinfold thickness was observed.

### 3.3. Principal Component Analysis of the Skinfolds Thickness Mostly Related to Metabolic Risks

**Figure 2:**
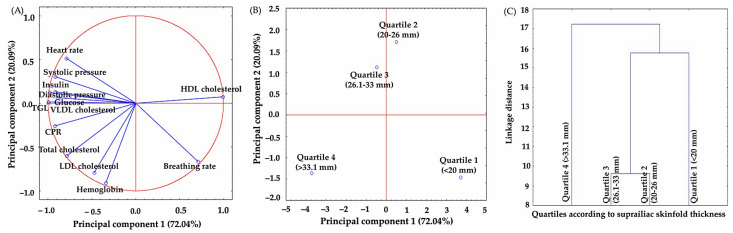
Principal component analysis (PCA) of the thickness of suprailiac skinfold. Relationship between clinical and biochemical parameters (**A**), distribution pattern of subjects according to their suprailiac skinfold thickness (**B**) and grouping (similarities or differences) dendrogram according to their suprailiac skinfold thickness (**C**). PCA was calculated without rotation, and the number of extracted factors was based on eigenvalues > 0.5 and explained variance > 90%.

**Figure 3:**
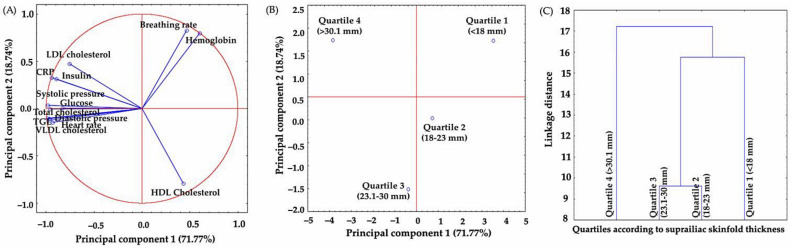
Principal components analysis (PCA) of the thickness of subscapular skinfold. Relationship between clinical and biochemical parameters (**A**), distribution pattern of subjects according to their suprailiac skinfold thickness (**B**) and grouping (similarities or differences) dendrogram according to their suprailiac skinfold thickness (**C**). PCA was calculated without rotation, and the number of extracted factors was based on eigenvalues > 0.5 and explained variance > 90%.

The principal component analysis is a statistical technique that calculates a new set of uncorrelated variables whose variances gradually decrease [22]. This study applied this statistical tool to identify associations between the suprailiac and subscapular skinfold thickness as well as some clinical and biochemical parameters (Figure 2 and Figure 3). The relationship between the suprailiac skinfold and some clinical and biochemical parameters of evaluated subjects is given in Figure 2A, accounting for 92.98% of the total variance in the first two principal components (PC1 72.04% and PC2 20.94%). In this context, PC1 (from negative to positive values) is characterized by a decrease in VLDL cholesterol (−0.99), triglycerides (−0.99), insulin (−0.98), ultrasensitive CRP (−0.92), systolic and diastolic pressure (−0.92 and −0.91), glucose (−0.89), heart rate (−0.79), and total cholesterol (−0.78) values as well as an increase in HDL cholesterol values (0.99). From the results in Figure 2B,C, quartiles are separated according to their suprailiac skinfold thickness, suggesting the existence of similitude among quartile 2 and quartile 3, where skinfold thickness ranges from 20 to 33 mm. In contrast, quartile 1 and quartile 4 are different due to their thickness differences.

Regarding subscapular skinfold thickness, Figure 3A shows that PC1 and PC2 explained 90.51% of the total variance (PC1 71.77% and PC2 18.74%), proceeding from negative to positive, in which VLDL cholesterol (−0.99), diastolic blood pressure (−0.99), systolic (−0.98), triglycerides (−0.97), glucose (−0.96), C-reactive protein (−0.94), insulin (−0.89), total cholesterol (−0.83) and LDL cholesterol (−0.75) are modified by the hemoglobin (0.60) content. Quartile 2 and quartile 3 have similar values; while quartile 1 and quartile 4 differ from one another; the latter is associated with the subscapular skinfold thickness (Figure 3B,C).

## 4. Discussion

Sedentary habits significantly affect people’s general health worldwide, increasing the prevalence and risk of non-communicable diseases such as diabetes, hypertension, hyperlipidemia, and obesity [23]. For a long time, the body mass index (BMI) was considered a good indicator of overweight as well as systemic and generalized obesity. However, this index does not consider the distribution of body adiposity or even its relationship with visceral adiposity, generating limitations in assessing cardiometabolic risks [24]. Furthermore, it has been observed that BMI is not sensitive to the fat distribution pattern during aging [25,26]. Different authors report that skinfold thickness measurement is recommended when a large group of participants are evaluated, and there is no access to suitable equipment, as skinfold results have shown better correlations than other methods [27,28,29]. Thus, skinfold thickness measurements could better predict total body fat in comparison to BMI [30]. Moreover, upper arm skinfolds were a more robust indicator of body fat percentage than BMI [31]. In this context, the present study provides elements to discuss the relationship between the distribution of body adiposity through skinfold thickness (bicipital, tricipital, subscapular, and suprailiac) measurements and the presence of cardiometabolic risks in sedentary and active adult populations.

In general, significant differences were observed between females and males in anthropometric, clinical, and biochemical parameters. However, it is documented that the gender difference exists due to musculoskeletal peculiarities and metabolic characteristics specific to each gender through hormones, molecular changes, and even cardiovascular illnesses; men have a more significant amount of lean mass, fast twitch muscle fibers, and muscle strength independent of exercise [32]. In the case of women, a high level of physical activity can slow weight gain [33], and physical training can induce changes in the skeletal muscle phenotype and modify nutrient reserves [32]. Also, it has been evidenced that women oxidize more lipids than men during moderate-intensity exercise [34]. Therefore, we could infer an impact on the body composition of women who exercise, being feasible to observe evident differences between women who were compared by physical activity.

Regarding BMI and fat percentage indicators, the population is generally overweight. However, differences were found by gender, including weight, height, percentage of fat, and circumferences, due to their physiological characteristics [35,36]. Men tend to be taller and have greater muscle mass, while women tend to store a more significant amount of subcutaneous adipose tissue, which is distributed mainly in the gluteal–femoral region [37]. It has been reported that women have a greater skinfold thickness than men [38], which is consistent with our findings, excluding the suprailiac fold, where men have a higher fat accumulation at the truncal level [39]. Contrary to expectations, the highest percentage of body fat was observed in physically active men than in non-active men. It is important to mention that the physical activity was self-reported, and no type, time, intensity, or length was recorded. In this context, it is possible that subjects included in this study recently started to engage in exercise [40]. Furthermore, if the physical activity performance is not accompanied by an intentional calorie deficit or adequate time and intensity, fat loss will be minimal [41]. Although the benefit of physical activity and regular exercise in regulating appetite and satiety has been documented, there are also reported actions showing compensatory mechanisms; these include a preference for the intake of sweet and high-fat foods in exercisers compared to non-exercisers [42].

The systolic and diastolic blood pressure values were higher in the male population. In this context, the majority of the population was middle-aged, and it is indicated that men have a higher incidence of hypertension than women who are less than 60 years old or even before women show menopausal symptoms, where hormones play a crucial role, especially estrogens, in reducing blood pressure levels by increasing nitric oxide synthase signaling [43]. In addition, lipid parameters such as triglycerides, total cholesterol, low-density cholesterol, and globular sedimentation volume, exhibited higher values in men than in women. This can be explained by the fact that the fat accumulated in men is located around the abdominal organs. This is known as visceral fat, which is located at the central level and is associated with elevated levels of triglycerides and free fatty acids [37]. In women, reduced HDL levels stand out and are consistent with other studies where it is mentioned that women have a severe tendency to lower HDL levels from 20 to 80 years old [44,45].

When comparing the effect of physical activity by gender, it was found that women who did not partake in physical activity had higher indices of fat, BMI, weight, circumference, and even bicipital skinfold compared to physically active women, which is an expected effect since exercise has favorable effects on these parameters [46,47]. Furthermore, active men reported lower insulin levels and higher body fat percentage, which can be explained by the body reconstitution favored by exercise; when performing physical activity, visceral fat is positively correlated with insulin levels, favoring insulin regulation [37,48,49,50].

Regarding body anthropometry, significant changes in some parameters (fat percentage, fat mass, systolic pressure, glucose, LDL cholesterol, fibrinogen, and insulin) were observed when the skinfolds (bicipital, tricipital, suprailiac, and subscapular) increased. It has been reported that body fat (through skinfold measurement) could be a harmful cardiovascular disease risk factor [50,51]. However, most studies relating skinfolds to metabolic risks have been conducted in children to assess subcutaneous adipose tissue [52]. Various skinfold predictions have been reported; the suprailiac skinfold has shown high predictive power for overweight and obesity in boys and girls [53]. The subscapular skinfold was strongly associated with triglyceride concentrations. In general, trunk skinfolds were associated with increases in triglycerides, low-density cholesterol, and insulin concentrations as well as with a decrease in high-density cholesterol; concerning limb skinfolds, and the weakest association was found in the tricipital skinfold [30].

Additionally, the sum of skinfold thickness (bicipital, tricipital, suprailiac, and subscapular) was associated with abdominal obesity, waist-to-height ratio, hyperglycemia, HOMA-IR, and C-reactive protein; data were consistent with previous studies in adolescents that reported associations between the sum of skinfolds with HOMA-IR, C-reactive protein, and blood pressure [54].

The increase in body adiposity also leads to increased blood pressure as observed in the present study. The systolic and diastolic blood pressure increased as the sum of skinfolds increased, which is consistent with previous studies conducted in children [55]. Moreover, a recent study found a strong association between subscapular skinfold thickness and the development of type 2 diabetes mellitus and arterial hypertension as well as an increased risk of developing type 2 diabetes mellitus [13]. Moreover, a recent study found a strong association between subscapular skinfold thickness and the development of type 2 diabetes mellitus and arterial hypertension as well as an increased risk of developing type 2 diabetes mellitus [13].

Regarding the association of skinfolds with the presence of metabolic risks, the highest correlation coefficient for abdominal obesity and cardiovascular risk by waist-to-height ratio was found in the subscapular skinfold, thus demonstrating the importance of body fat distribution, since an increase in total cholesterol and LDL cholesterol levels in subjects with more significant subscapular obesity has been associated with increased risk of developing hypertension and type 2 diabetes, which is consistent with the results found for insulin resistance and blood pressure levels [48]. In addition, this fold has been previously associated with an increased risk of developing arterial hypertension and type 2 diabetes, which is consistent with the results found regarding insulin resistance and blood pressure values [13,56].

In this context, the subscapular skinfold in conjunction with the suprailiac skinfold has been associated with elevated C-reactive protein levels indicative of inflammatory processes [57], which is consistent with our findings. The strongest correlations were shown with both truncal folds, where the relationship with visceral fat is direct [58]. Suprailiac and subscapular skinfolds showed the strongest association with metabolic risks. It has been previously reported that these skinfolds, in particular, act as proxies for central adiposity during early childhood [17]. It is believed that these skinfolds may also increase according to their inheritance in a study where truncal fat was analyzed concerning the presence or not of parents with diabetes [59]. In a previous study of adolescent males, the suprailiac skinfold was found to be the best determinant of the metabolic risk score [60]. Concerning the skinfolds of the upper extremities, only the bicipital fold obtained the highest correlation with hyperglycemia in the evaluated subjects, coinciding with reports that associate it with a higher risk of type 2 diabetes [13,56].

On the other hand, a moderate negative association was found between the tricipital skinfold and hemoglobin. Moreover, a decrease in subscapular skinfold thickness was found to correlate with an increase in hemoglobin concentration. This connection was reported in adolescents, specifically when the sum of skinfold thickness was considered [61]. This outcome could be attributed to the reduced oxygen uptake observed in obese individuals, which might lead to diminished blood flow. Consequently, this reduced blood flow could contribute to decreased levels of red blood cells and hemoglobin [61,62].

Regarding the analysis of physical activity, no differences were found in the tricipital, subscapular, or suprailiac skinfolds. The only differences were observed in the thickness of the bicipital skinfold, which was lower in those subjects who reported performing some physical activity, as it produced an energy expenditure when conducting musculoskeletal movement [63]. This characteristic has been demonstrated in several studies where physical activity and exercise show a direct effect with weight loss and body fat [64,65], which could be explained by the darkening of white adipose tissue mediated by exercise through different physiological processes, involving the activation of some endorphins, the role of lipolysis, the activation of reactive oxygen species, the production of some metabolites such as ketone bodies, lactate, and succinate pathways [66].

## 5. Limitations

Among the limitations of this cross-sectional study, it is important to highlight that the type, intensity, and duration of physical activity reported by subjects were not measured. Additionally, the study did not identify the duration for which subjects had been engaging in physical activity. Moreover, the age range of the studied population also exhibited heterogeneity, spanning from 18 to 79 years; notably, 73.5% of the participants were above 40 years old. In this context, it is expected that advancing age could coincide with a tendency for muscle mass reduction and an increase in body fat. Furthermore, it is necessary to emphasize that this study was conducted within a specific Mexican city’s population, which may restrict the generalization of the findings to the population.

## 6. Conclusions

Although the study was conducted in individuals with no apparent disease or conditions, systolic blood pressure and waist circumference were found to be high. This result is a direct indicator of visceral fat related to cardiometabolic risks. The suprailiac and subscapular skinfolds showed the most significant association with probable cardiometabolic risks in the evaluated adult population, mainly in the sedentary population and the latter in the physically active population. Therefore, these skinfolds can be considered in assessing the adult population for early cardiometabolic risks, even when evaluating a healthy and active population. Further multicenter and longitudinal studies in apparently healthy subjects are required to validate the association of skinfold and cardiometabolic risks found in this study. Moreover, further variables should be included in future studies such as ethnicities, races, and ages as well as a detailed examination of physical activity that considers the type, intensity, and duration of activity per day and month that subjects have been exercising.

## Figures and Tables

**Table 1:** Evaluation methods and reference parameters for the determination of cardiometabolic risks.

Variable	Method	Parameter	Ref.
Abdominal obesity	Measure the narrowest part of the torso	>90 cm—men >85 cm—women	[18]
Waist-to-height ratio	Divide waist circumference by height.	>0.5	[18]
Insulin resistance	HOMA-IR: Fasting Insulin (µU/mL) × Fasting Glucose (mmol/L)/22.5	>2.5	[19]
Hyperglycemia	Enzymatic colorimetric method (Biosystems^®^, Barcelona, Spain)	>100 mg/dL	[20]
Hypoalphalipoproteinemia	Enzymatic colorimetric method (Biosystems^®^, Barcelona, Spain) Hight-density cholesterol	<40 mg/dL men <50 mg/dL women	[20]
Hypertriglyceridemia	Enzymatic colorimetric method (Biosystems^®^, Barcelona, Spain) Triglycerides	>150 mg/dL	[20]
Risk for elevated blood pressure	Automatic measurement in the upper left arm with the digital baumanometer device (Omron^®^, Kyoto Japan) in supine position after 5 min of rest.	>130 systolic m/Hg >85 diastolic mm/Hg	[21]

HOMA IR: homeostatic model assessment for insulin resistance.

**Table 2:** Anthropometric, clinical, and biochemical characteristics by sex and physical activity.

Variable	Female Total n = 616	Male Total n = 330	*p*-Value	Female PA n = 335	Female NA n = 281	*p*-Value	Male PA n = 184	Male NA n = 146	*p*-Value
	Mean ± SD	Mean ± SD		Mean Male Sex ± SD	Mean ± SD		Mean ± SD	Mean ± SD	
Age (years)	43.77 ± 11.63	43.61 ± 12.78	0.850	43.24 ± 12.26	44.39 ± 10.82	0.222	43.20 ± 12.55	44.12 ± 13.10	0.513
Weight (kg)	68.88 ± 13.44	80.73 ± 13.81	<0.001 *	67.62 ± 12.92	70.39 ± 13.92	0.011 *	81.51 ± 14.04	79.74 ± 13.51	0.249
Height (m)	1.58 ± 0.06	1.70 ± 0.07	<0.001 *	1.58 ± 0.06	1.58 ± 0.06	0.746	1.70 ± 0.71	1.71 ± 0.07	0.232
Body Mass Index (kg/m^2^)	27.42 ± 5.21	27.77 ± 4.33	0.264	26.95 ± 5.04	27.99 ± 5.35	0.013 *	28.16 ± 4.32	27.29 ± 4.30	0.070
Fat by bioimpedance (%)	35.03 ± 6.91	25.21 ± 6.77	<0.001 *	34.30 ± 7.46	35.89 ± 6.10	0.004 *	26.09 ± 6.58	24.11 ± 6.87	0.008 *
Waist (cm)	87.79 ± 13.14	96.01 ± 11.55	<0.001 *	86.55 ± 12.67	89.27 ± 13.54	0.010 *	97.02 ± 11.28	94.73 ± 11.79	0.074
Hip (cm)	104.56 ± 10.45	102.72 ± 8.29	<0.001 *	103.53 ± 10.49	105.78 ± 10.99	0.008 *	103.37 ± 8.77	101.92 ± 7.59	0.114
Skinfold bicipital (mm)	20.83 ± 9.34	15.35 ± 9.11	<0.001 *	19.89 ± 9.40	21.94 ± 9.16	0.007 *	15.41 ± 9.03	15.30 ± 9.23	0.915
Skinfold tricipital (mm)	27.93 ± 8.23	20.55 ± 8.67	<0.001 *	27.39 ± 8.22	28.56 ± 8.37	0.080	20.81 ± 8.40	20.21 ± 9.01	0.525
Skinfold suprailiac (mm)	27.59 ± 9.33	25.58 ± 10.49	0.004 *	27.21 ± 9.11	28.03 ± 9.59	0.277	25.90 ± 10.63	25.17 ± 10.36	0.530
Skinfold subscapular (mm)	25.20 ± 9.45	23.09 ± 8.95	0.001 *	25.11 ± 9.39	25.31 ± 9.55	0.793	23.02 ± 9.14	23.19 ± 9.14	0.857
Fold sum (mm)	101.53 ± 29.56	84.58 ± 29.91	<0.001 *	99.60 ± 29.28	103.84 ± 29.77	0.076	85.14 ± 29.39	83.87 ± 30.64	0.702
Systolic pressure (mm/Hg)	115.05 ± 15.36	119.21 ± 15.16	<0.001 *	114.90 ± 15.24	115.22 ± 15.53	0.801	118.67 ± 13.90	119.89 ± 16.64	0.469
Diastolic pressure (mm/Hg)	74.48 ± 10.22	77.64 ± 10.39	<0.001 *	74.36 ± 9.74	74.61 ± 10.79	0.760	77.33 ± 9.72	78.03 ± 11.20	0.545
Heart rate (beats/min)	69.01 ± 7.47	68.88 ± 7.56	0.798	68.64 ± 7.41	69.45 ± 7.54	0.185	68.97 ± 7.88	68.76 ± 7.16	0.800
Breathing rate (breaths/min)	17.19 ± 2.33	17.21 ± 2.48	0.885	17.32 ± 2.33	17.03 ± 2.32	0.119	17.23 ± 2.57	17.18 ± 2.30	0.873
Hemoglobin (g/dL)	13.45 ± 1.30	15.03 ± 1.44	<0.001 *	13.50 ± 1.25	13.39 ± 1.35	0.307	14.96 ± 1.41	15.13 ± 1.48	0.274
Hematocrit (mm/dL)	42.14 ± 3.48	46.94 ± 3.57	<0.001 *	42.18 ± 3.42	42.10 ± 3.55	0.771	46.94 ± 3.61	46.94 ± 3.55	0.996
Glucose (mg/dL)	94.51 ± 22.79	95.75 ± 20.13	0.407	93.84 ± 24.27	95.30 ± 20.91	0.430	95.54 ± 18.48	96.01 ± 22.10	0.832
Total cholesterol (mg/dL)	183.87 ± 41.45	193.34 ± 49.53	0.003 *	181.08 ± 41.19	187.19 ± 41.59	0.069	192.62 ± 48.24	194.25 ± 51.27	0.767
HDL cholesterol (mg/dL)	44.16 ± 18.72	40.75 ± 18.04	0.007 *	43.22 ± 18.76	45.27 ± 18.63	0.175	40.44 ± 17.97	41.15 ± 18.17	0.723
LDL cholesterol (mg/dL)	113.78 ± 41.22	118.21 ± 48.03	0.156	112.40 ± 40.93	115.43 ± 41.59	0.365	118.45 ± 45.44	117.93 ± 51.27	0.921
VLDL cholesterol (mg/dL)	25.99 ± 13.35	34.47 ± 20.81	<0.001 *	25.53 ± 14.22	26.55 ± 12.24	0.347	34.11 ± 21.01	34.93 ± 20.63	0.724
Triglycerides (mg/dL)	129.98 ± 66.76	172.98 ± 104.81	<0.001 *	127.67 ± 71.11	132.74 ± 61.18	0.347	170.55 ± 105.06	176.04 ± 104.76	0.637
Globular sedimentation velocity (mm/h)	14.84 ± 10.05	11.80 ± 9.59	<0.001 *	15.43 ± 10.57	14.13 ± 9.35	0.107	12.73 ± 10.94	10.62 ± 7.43	0.047*
Fibrinogen (mg/dL)	327.75 ± 86.53	328.63 ± 85.15	0.880	322.57 ± 85.43	333.92 ± 87.58	0.105	330.58 ± 86.42	326.18 ± 83.74	0.641
Insulin (U/mL)	10.24 ± 10.15	10.47 ± 10.28	0.735	10.20 ± 11.66	10.29 ± 8.01	0.909	9.39 ± 6.53	11.84 ± 13.51	0.031*
Ultrasensitive CRP (mg/L)	1.89 ± 3.04	2.04 ± 3.05	0.483	1.91 ± 3.49	1.88 ± 2.42	0.901	1.99 ± 3.09	2.09 ± 3.01	0.783

Student´s *t*-test, level of significance * *p* < 0.05. SD: Standard Deviation, PA: Physical Activity, NA: No Physical Activity, HDL: High-Density Lipoprotein, LDL: Low-Density Lipoprotein, VLDL: Very-Low-Density Lipoprotein, CRP: C-Reactive Protein.

**Table 3:** Clinical and biochemical characteristics by skinfold thickness.

Variables	Quartile 1 <74.89 mm n = 237	Quartile 2 74.90–93.99 mm n = 237	Quartile 3 94.00–114.99 mm n = 236	Quartile 4 >114.10 mm n = 236	*p*-Value
	Mean ±SD	Mean ±SD	Mean ±SD	Mean ±SD	
Systolic pressure (mm/Hg)	113.76 ± 14.28	117.41 ± 15.74	116.93 ± 17.63	117.89 ± 13.37	0.015 ^a,b^
Diastolic pressure (mm/Hg)	73.35 ± 9.77	76.16 ± 10.24	76.25 ± 11.28	76.58 ± 9.90	0.002 ^a,b,c^
Heart rate (beats/min)	67.16 ± 7.70	69.03 ± 8.06	69.92 ± 6.99	69.75 ± 6.88	<0.001 ^a,b,c^
Breathing rate (breaths/min)	17.77 ± 2.40	16.97 ± 2.54	17.08 ± 2.23	16.95 ± 2.20	<0.001 ^a,b,c^
Hemoglobin (g/dL)	14.51 ± 1.65	13.83 ± 1.49	13.84 ± 1.52	13.81 ± 1.41	<0.001 ^a,b,c^
Hematocrit (mm/dL)	44.66 ± 4.36	43.58 ± 4.43	43.60 ± 4.12	43.42 ± 3.71	0.004 ^a,b,c^
Glucose (mg/dL)	91.16 ± 18.55	91.92 ± 15.40	96.64 ± 24.85	100.55 ± 26.74	<0.001 ^b,c,d^
Total cholesterol (mg/dL)	184.57 ± 48.36	184.42 ± 41.76	188.61 ± 44.68	191.47 ± 43.63	0.249
HDL cholesterol (mg/dL)	42.43 ± 19.39	42.14 ± 18.71	44.62 ± 19.23	42.87 ± 16.91	0.469
LDL cholesterol (mg/dL)	114.71 ± 46.65	114.91 ± 44.05	114.97 ± 42.72	116.85 ± 41.57	0.947
VLDL cholesterol (mg/dL)	27.55 ± 18.40	27.43 ± 13.69	29.09 ± 16.37	31.82 ± 18.15	0.015 ^b,d^
Triglycerides (mg/dL)	138.63 ± 93.49	137.13 ± 68.45	145.47 ± 81.93	159.09 ± 90.77	0.019 ^b,d^
Globular sedimentation velocity (mm/h)	11.49 ± 8.53	12.82 ± 8.07	14.42 ± 9.62	16.43 ± 12.53	<0.001 ^b,c,d^
Fibrinogen (mg/dL)	300.97 ± 76.89	327.79 ± 82.96	330.25 ± 83.48	353.58 ± 92.27	<0.001 ^a,b,c,d,e^
Insulin (U/mL)	8.66 ± 9.03	10.38 ± 11.02	10.66 ± 10.48	11.60 ± 9.95	0.016 ^b^
C-reactive protein (mg/L)	1.31 ± 3.36	1.82 ± 2.73	1.75 ± 2.28	2.90 ± 3.43	<0.001 ^b,d,e^
Fat by bioimpedance (%)	24.46 ± 7.73	31.91 ± 6.75	33.43 ± 6.81	36.68 ± 6.67	<0.001 ^a,b,c,d,e^
Fat mass (kg)	16.92 ± 7.77	22.41 ± 7.38	24.06 ± 7.40	28.42 ± 9.30	<0.001 ^a,b,c,d,e^

Subjects were distributed in quartiles according to their skinfold thickness and ANOVA *t*-test. Post hoc Tukey was performed. A comparison between quartiles was carried out as follows: ^a^ Quartile 1 vs. Quartile 2, ^b^ Quartile 1 vs. Quartile 4, ^c^ Quartile 1 vs. Quartile 3, ^d^ Quartile 2 vs. Quartile 4, ^e^ Quartile 3 vs. Quartile 4.

**Table 4:** Association of metabolic risks concerning skinfold thickness in sedentary and active adults.

Cardiometabolic Risk		Bicipital (mm)		Tricipital (mm)		Suprailiac (mm)		Subscapular (mm)	
% (number of cases)		(19.7) NA	(18.3) PA	(25.7) NA	(25.1) PA	(27.1) NA	(26.7) PA	(24.6) NA	(24.4) PA
Cardiovascular by waist–height index 76.8% (n = 727)	Cramer´s V	(23.6) n = 338 0.218 **	(21.1) n = 389 0.229 **	(28.7) n = 338 0.269 **	(26.6) n = 389 0.263 **	(30.5) n = 338 0.400 **	(28.7) n = 389 0.397 **	(27.6) n = 338 0.444 **	(26.6) n = 389 0.452 **
Hypoalphalipoproteinemia 62.1% (n = 587)						(28.5) n = 251 0.179 *			(24.9) n = 336 0.151 *
Abdominal obesity 53.5% (n = 506)		(22.0) n = 232 0.224 **	(20.3) n = 274 0.248 **	(28.3) n = 232 0.257 **	(27.3) n = 274 0.233 **	(31.3) n = 232 0.459 **	(30.6) n = 274 0.434 **	(28.5) n = 232 0.469 **	(28.3) n = 274 0.466 **
Hypertriglyceridemia 31.7% (n = 300)						(29.4) n = 145 0.184 *	(29.1) n = 155 0.164 *	(26.3) n = 145 0.141 *	(26.8) n = 155 0.168 *
Hyperglycemia 26.6% (n = 252)		(23.8) n = 118 0.257 **	(20.8) n = 134 0.198 **	(28.79 n = 118 0.190 **	(26.4) n = 134 0.143 *	(30.7) n = 118 0.196 **	(28.4) n = 134 0.130 *	(28.0) n = 118 0.218 **	(26.2) n = 134 0.136 **
Hypertension 24.3% (n = 300)			(19.4) n = 121 0.141*		(26.5) n = 121 0.136*				(26.4) n = 121 0.146 *
Insulin resistance by HOMA-IR index 22.3% (n = 211)		(22.1) n = 109 0.152 *				(31.6) n = 109 0.292 **	(30.2) n = 102 0.179 **	(29.2) n = 109 0.237 **	(27.7) n = 102 0.180 *

Ji2 test * *p* < 0.05, ** *p* < 0.001. mm = millimeters. NA = No Physical Activity; PA = Physical Activity.

**Table 5:** Correlation between metabolic risk and skinfold thickness.

Cardiometabolic Risk			Bicipital	Tricipital	Suprailiac	Subscapular	Fold Sum
Abdominal obesity	Correlation coefficient	S	r = 0.230 **	r = 0.267 **	r = 0.457 **	r = 0.473 **	r = 0.433 **
Waist/Height ratio		S	r = 0.232 **	r = 0.266 **	r = 0.408 **	r = 0.463 **	r = 0.408 **
Insulin resistance		S	r = 0.115 **	r = 0.109 **	r = 0.233 **	r = 0.211 **	r = 0.205 **
Hyperglycemia		S	r = 0.210 **	r = 0.144 **	r = 0.163 **	r = 0.164 **	r = 0.204 **
Glucose		P	r = 0.159 **	r = 0.129 **	r = 0.123 **	r = 0.177 **	r = 0.181 **
C-reactive protein		P	r = 0.133 **	r = 0.123 **	r = 0.214 **	r = 0.213 **	r = 0.210 **
Heart rate		P	r = 0.132 **	r = 0.123 **		r = 0.124 **	r = 0.137 **
Systolic pressure		P			r = 0.125 **	r = 0.166 **	r = 0.134 **
Diastolic pressure		P			r = 0.111 **	r = 0.162 **	r = 0.107 **
Systemic arterial hypertension		S				r = 0.106 **	
Breathing rate		S	r = −0.155 **	r = −0.149 **			r = −0.122 **
Hemoglobin		P	r = −0.218 **	r = −0.318 **			r = −0.155 **
Hypertriglyceridemia		S			r = 0.168 **	r = 0.157 **	r = 0.122 **

P = Pearson’s correlation, S = Spearman’s correlation, r = Rho, ** *p* < 0.001.

## Data Availability

The dataset used and/or analyzed during the current study are available from the corresponding author on reasonable request.

## Supplementary Materials

The following supporting information can be downloaded at: https://www.mdpi.com/article/10.3390/jpm13091326/s1, Table S1. Anthropometric, clinical and biochemical characteristics by sex.
