# Supplementary material for: Skinfold Thickness as a Cardiometabolic Risk Predictor in Sedentary and Active Adult Populations
Source: J Pers Med. 2023 Aug 29;13(9):1326. doi: 10.3390/jpm13091326 (PMC10532477; doi:10.3390/jpm13091326)
Supplement: Supplementary file 1 [file jpm-13-01326-s001.zip › jpm-2557187-supplementary.pdf]

Table S1. Anthropometric, clinical and biochemical characteristics by sex

Variables	Total n=946 Mean ±SD	Female n=616 Mean ±SD	Male n=330 Mean ±SD	*p* Value
Age (years)	43.72 ± 12.09	43.77 ± 11.63	43.61 ± 12.78	*0.850*
Weight (kg)	73.02 ± 14.69	68.88 ± 13.44	80.73 ± 13.81	*<0.001**
Height (Mts)	1.62 ± 0.09	1.58 ± 0.06	1.70 ± 0.07	*<0.001**
Body Mass Index (kg/m^2)	27.55 ± 4.92	27.42 ± 5.21	27.77 ± 4.33	*0.264*
Grasa (%) Bioimpedance	31.61 ± 8.31	35.03 ± 6.91	25.21 ± 6.77	*<0.001**
Waist (cm)	90.66 ± 13.19	87.79 ± 13.14	96.01 ± 11.55	*<0.001**
Hip (cm)	103.92 ± 9.82	104.56 ± 10.45	102.72 ± 8.29	*<0.001**
Skin fold Bicipital (mm)	18.92 ± 9.61	20.83 ± 9.34	15.35 ± 9.11	*<0.001**
Skin fold Tricipital (mm)	25.35 ± 9.09	27.93 ± 8.23	20.55 ± 8.67	*<0.001**
Skin fold Supraileac (mm)	26.89 ± 9.79	27.59 ± 9.33	25.58 ± 10.49	*0.004**
Skin fold Subscapular (mm)	24.47 ± 9.33	25.20 ± 9.45	23.09 ± 8.95	*0.001**
Fold sum (mm)	95.62 ± 30.75	101.53 ± 29.56	84.58 ± 29.91	*<0.001**
Sistolic pressure (mm/Hg)	116.49 ± 15.41	115.05 ± 15.36	119.21 ± 15.16	*<0.001**
Diastolic pressure (mm/Hg)	75.58 ± 10.39	74.48 ± 10.22	77.64 ± 10.39	*<0.001**
Hear rate (beats/m)	68.96 ± 7.50	69.01 ± 7.47	68.88 ± 7.56	*0.798*
Breathing rate (breaths/m)	17.19 ± 2.37	17.19 ± 2.33	17.21 ± 2.48	*0.885*
Hemoglobin (g/dL)	14.00 ± 1.55	13.45 ± 1.30	15.03 ± 1.44	*<0.001**
Hematocrit (mm/dL)	43.82 ± 4.19	42.14 ± 3.48	46.94 ± 3.57	*<0.001**
Glucose (mg/dL)	94.94 ± 21.89	94.51 ± 22.79	95.75 ± 20.13	*0.407*
Total Cholesterol (mg/dL)	187.17 ± 44.64	183.87 ± 41.45	193.34 ± 49.53	*0.003**
HDL Cholesterol (mg/dL)	42.97 ± 18.54	44.16 ± 18.72	40.75 ± 18.04	*0.007**
LDL Cholesterol (mg/dL)	115.33 ± 43.75	113.78 ± 41.22	118.21 ± 48.03	*0.156*
VLDL Cholesterol (mg/dL)	28.95 ± 16.83	25.99 ± 13.35	34.47 ± 20.81	*<0.001**
Tryglicerides (mg/dL)	144.98 ± 84.53	129.98 ± 66.76	172.98 ± 104.81	*<0.001**
Total leukocytes (mm^3)	125.18 ±182.79	130.13 ± 189.32	115.92 ± 169.81	*0.239*
Platelets (mcL)	254.25 ± 62.66	255.58 ± 62.66	251.76 ± 62.67	*0.371*
Globular sedimentation velocity (mm)	13.78 ± 9.99	14.84 ± 10.05	11.80 ± 9.59	*<0.001**
Fibrionogen (mg/dL)	328.06 ± 80.01	327.75 ± 86.53	328.63 ± 85.15	*0.880*
Insulin (U/ml)	10.32 ± 10.19	10.24 ± 10.15	10.47 ± 10.28	*0.735*
Ultrasensitive CRP (mg/L)	1.95 ± 3.04	1.89 ± 3.04	2.04 ± 3.05	*0.483*

Student´s t-test, level of significance *$p<0.05$. HDL: Hight Density Lipoprotein, LDL: Low Density Lipoprotein, VLDL: Very Low Density Lipoprotein, CRP: C-Reactive Protein.
